# Low incidence of multidrug-resistant bacteria and nosocomial infection due to a preventive multimodal nosocomial infection control: a 10-year single centre prospective cohort study in neurocritical care

**DOI:** 10.1186/s12883-018-1031-6

**Published:** 2018-03-07

**Authors:** Vera Spatenkova, Ondrej Bradac, Daniela Fackova, Zdenka Bohunova, Petr Suchomel

**Affiliations:** 1 Neurocenter, Neurointensive Care Unit, Regional Hospital, Husova 357/10, Regional Hospital, 46063 Liberec, Czech Republic; 2 Department of Neurosurgery, Military University Hospital and First Medical School, Charles University, Prague, Czech Republic; 3 Department of Clinical microbiology and immunology, Antibiotic Centre, Regional Hospital, Liberec, Czech Republic; 4 Neurocenter, Department of Neurosurgery, Regional Hospital, Liberec, Czech Republic

**Keywords:** Neurocritical care, Nosocomial infections, Multidrug-resistant bacteria, Outcome, Preventive protocol

## Abstract

**Background:**

Nosocomial infection (NI) control is an important issue in neurocritical care due to secondary brain damage and the increased morbidity and mortality of primary acute neurocritical care patients. The primary aim of this study was to determine incidence of nosocomial infections and multidrug-resistant bacteria and seek predictors of nosocomial infections in a preventive multimodal nosocomial infection protocol in the neurointensive care unit (NICU). The secondary aim focused on their impact on stay, mortality and cost in the NICU.

**Methods:**

A10-year, single-centre prospective observational cohort study was conducted on 3464 acute brain disease patients. There were 198 (5.7%) patients with nosocomial infection (wound 2.1%, respiratory 1.8%, urinary 1.0%, bloodstream 0.7% and other 0.1%); 67 (1.9%) with Extended spectrum beta-lactamase (ESBL); 52 (1.5%) with Methicillin-resistant Staphylococcus aureus (MRSA), nobody with Vancomycin-resistant enterococcus (VRE). The protocol included hygienic, epidemiological status and antibiotic policy. Univariate and multivarite logistic regression analysis was used for identifying predictors of nosocomial infection.

**Results:**

From 198 NI patients, 153 had onset of NI during their NICU stay (4.4%; wound 1.0%, respiratory 1.7%, urinary 0.9%, bloodstream 0.6%, other 0.1%); ESBL in 31 (0.9%) patients, MRSA in 30 (0.9%) patients. Antibiotics in prophylaxis was given to 63.0% patients (59.2 % for operations), in therapy to 9.7% patients. Predictors of NI in multivariate logistic regression analysis were airways (OR 2.69, 95% CI 1.81-3.99, *p*<0.001), urine catheters (OR 2.77, 95% CI 1.00-7.70, *p*=0.050), NICU stay (OR 1.14, 95% CI 1.12-1.16, *p*<0.001), transfusions (OR 1.79, 95% CI 1.07-2.97, *p*=0.025) antibiotic prophylaxis (OR 0.50, 95% CI 0.34-0.74, *p*<0.001), wound complications (OR 2.30, 95% CI 1.33-3.97, *p*=0.003). NI patients had longer stay (*p*<0.001), higher mortality (*p*<0.001) and higher TISS sums (*p*<0.001) in the NICU.

**Conclusions:**

The presented preventive multimodal nosocomial infection control management was efficient; it gave low rates of nosocomial infections (4.2%) and multidrug-resistant bacteria (ESBL 0.9%, MRSA 0.9% and no VRE). Strong predictors for onset of nosocomial infection were accesses such as airways and urine catheters, NICU stay, antibiotic prophylaxis, wound complications and transfusion. This study confirmed nosocomial infection is associated with worse outcome, higher cost and longer NICU stay.

## Background

Nosocomial infections (NI) are still an important issue in neurocritical care due to secondary brain damage and the increased morbidity and mortality of primary acute neurocritical care patients [1–5]. NI is associated with higher antibiotic consumption, thereby worsening the epidemiological situation in the intensive care unit by increasing the occurrence of multidrug-resistant bacteria [6]. For these reasons, they have a significant economic impact because they prolong stay [7–10] in the neurointensive care unit (NICU) and the higher frequency of diagnostic and therapeutic processing significantly raises healthcare costs.

Nosocomial infections can be caused by many risk factors, not all of which have been fully investigated. However, keeping a hygienic and epidemiological regime of critical care [11–13] and the rational use of antibiotics makes a significant impact [14, 15].

The primary aim of this study was to determine incidence of nosocomial infections and multidrug-resistant bacteria and seek predictors of nosocomial infections in a preventive multimodal nosocomial infection protocol in our neurocritical care. The secondary aim focused on their impact on stay, mortality and cost in the NICU.

## Method

### Study design and setting

A monocentric 10-year observation prospective cohort study was conducted in the entire population of 3464 patients with acute brain disease, admitted to an eight-bed, adult neurological and neurosurgical intensive care unit in the Neurocenter of the 900-bed Regional Hospital with a catchment area of approximately half a million people. The study was performed in the NICU, which consists of four different rooms: one room with one bed, two rooms with two beds and one room with three beds. The study was approved by the Liberec hospital Ethics Committees for Multicentric Clinical Trials.

We prospectively examined the following determined demographic and clinical parameters in our local NICU: brain diagnosis, type of admission (primary, secondary to 24 hours and after 24 hours; acute or planned; rehospitalisation), admission and overall Therapeutic Intervention Scoring System (TISS), admission Glasgow Coma Scale (GCS), admission Acute Physiology and Chronic Health Evaluation (APACHE) II score, length of stay in the NICU, mortality in the NICU, Glasgow Outcome Scale (GOS) upon discharge from the NICU, C-reactive protein (CRP), operations (amount, day of hospital and NICU hospitalisation, acute or planned, reoperation, time and type of operation), American Society of Anesthesiologists (ASA) Score, drainage, airways, mechanical ventilation, catheters (artery, central venous, urine) and tubes, administration of corticoids, transfusions, ulcer prophylaxis and diabetes mellitus.

### Preventive multimodal nosocomial infection protocol

In the preventive multimodal nosocomial infection protocol, we categorised hygienic and epidemiological status and antibiotic policy.

### Hygienic and epidemiological regime

The basis of the hygienic and epidemiological regime in our preventive multimodal protocol consisted of cleanliness, disinfection, sterilisation, barrier patient care techniques, the separation of clean and contaminated procedures and the regular monthly exchange of disinfectants. We categorised principles for staff, patients and facilities.

#### 1/Staff and visitors

The foremost part of this protocol was maintaining the hygiene and disinfection of all staff members’ hands before and after care for each patient, enabled by the bottled disinfectant provided at each entrance and each bed. This rule was also required for visitors. Staff members were not allowed to wear jewellery or watches on their hands and had to keep their fingernails cut short. Internal staff had to wear new, clean, special NICU clothing every day, a protective coat when outside the NICU, and masks, surgical caps and gowns when caring for isolated patients or during invasive medical procedures. Aprons were worn while washing patients. External staff as well as visitors wore surgical gowns, but not overshoes, and only 2 family members were allowed in the patient’s room at a time.

#### 2/Patients

Care of the patient was performed on the principle of barrier care techniques. Tools for individual patients including disinfection, stethoscopes, thermometers and washing aids were available by each bed. Patients were washed twice a day with liquid soap. Disinfection soap was used only before entering the operating theatre. Oral hygiene included cleaning teeth with our special toothbrushes with chlorhexidine and subglottic secretion drainage, after washing, the patient’s body was rubbed with a non-allergic cream. Patients’ clothes and bedding were changed twice a day. Dirty laundry was put in special sacks rather than dropped freely on the floor.

Basic principles of care for drainage, catheters, infusion, suction from the airway, breathing circuit sets, tubes included: 1/single-use products, 2/closed systems, 3/the minimum necessary duration, 4/minimal and only necessary disconnection, using the port system, 5/the regular (peripheral venous catheters, all infusion sets, connecting tubes and ports) and irregular (central venous catheters, endotracheal tubes and tracheostomy) exchange of all these tubes and catheters was made according to the exchange protocol. Invasive procedures included the sterile insertion of systems and regularly exchanged, fully covering and constantly dry sterile wound covers. Furthermore, the protocol included the hourly monitoring of residual gastric volume.

The protocol included the regular microbiological screening of nose, throat, trachea, skin, urine and rectum from admission and then every three days, as well as every catheter except the peripheral venous for the timely detection of multidrug-resistant bacteria extended spectrum beta-lactamases (ESBL) or methicillin-resistant Staphylococcus aureus (MRSA) or Vancomycin-resistant enterococcus (VRE).

Patients with an infection or with multidrug-resistant bacteria ESBL and MRSA were completely isolated.

#### 3/Facilities

Daily cleaning with disinfection of surfaces including the bed, monitors, and other equipment around the bed, door handles and floors was conducted three times a day. Walls were cleaned once a day for the isolated patients, otherwise once a week. Each room had its own bucket for surfaces and walls. The floors were mopped using a system of two buckets and a cloth, with each room having its own. All cupboards containing materials and medical equipment were cleaned with disinfectant once a week. Waste was sorted and disposed of using specially marked plastic containers and sacks. After the patient was discharged, the bed was completely disinfected. The room was painted with a washable coating once a year.

### Antibiotic policy

The protocol included the monitoring of antibiotics in a local computer database. Antibiotic policy was implemented in close cooperation with the antibiotic centre and intended to keep the rational antibiotic policy aim of eliminating the overuse of antibiotics, especially those not used during bacterial pathogeny colonisation. The indications for using prophylactic antibiotics were surgical procedures (operation, external ventricular and lumbar drainage, intracranial sensors), liquorrhoea and aspiration. The protocol required maintaining dose and timing before the operation, perioperative administration for lengthy operations, and the non-prolongation of antibiotic administration after the operation or drainage or implantation of sensors. Empiric antibiotic therapy was to start after samples were taken for microbiological examination to enable their administration according to culture and sensitivity.

### Nosocomial infection

Infections were identified according to clinical symptoms such as fever, bacterial pathogens from secretions, liquor, urine, wounds, catheters, haemoculture with a defined microbiology colony count, imaging methods, biochemical and haematological laboratory tests. Nosocomial infections were defined as infections starting after two calendar days in the hospital. We identified nosocomial infections in 198 patients (5.7%). There were more wound infections (2.1%), than respiratory (1.8%), urinary (1.0%), bloodstream (0.7%) and others (0.1%).

### Statistical analysis

Parametric *t*-tests or non-parametric Mann-Whitney U tests were used for comparison of continuous variables. Comparison of categorical parameters was carried out using Chi-square or Fisher tests as appropriate. Univariate logistic regression was used for identifying prognostic factors of NI. Factors from univarite analysis with level of significance defined as *p* <0.1 were used for multivarite regression analysis, factors with *p* value <0.1 were left in the model. *P*–values of less than 0.05 were considered significant. STATISTICA 13.2 (TIBCO Software Inc., Palo Alto, CA, USA) software was used for statistical analyses. The control group was defined as patients without nosocomial infections.

## Results

We did not find any demographic differences such as age, gender, weight or body mass index between the NI group and the control group, as can be seen in Table 1. However, there was a difference in diagnosis, more patients with stroke and hydrocephalus had more NI than those with other diagnoses. According to the scoring system, patients with nosocomial infection upon admission had significantly lower GCS scale and higher APACHE II. Prognostic parameters were also significantly higher in the NI patients group. They stayed in the NICU longer, had higher mortality and worse Glasgow Coma Scale upon discharge. They were also more expensive economically, and had significantly higher total TISS.

**Table 1:** Demographic and clinical data of population of patients with acute brain disease, with or without nosocomial infection

Parameter	Unit	Total population	NI group	Control group	*p* value
Number total	pts	3464 (100%)	198 (5.7%)	3266 (94.3%)	
January	pts	327 (9.4%)	7 (3.6%)	310 (9.5%)	
February	pts	249 (7.2%)	19 (9.6%)	230 (7.0%)	
March	pts	267 (7.7%)	19 (9.6%)	248 (7.6%)	
April	pts	305 (8.8%)	13 (6.6%)	292 (8.9%)	
May	pts	269 (7.8%)	21 (10.6%)	248 (7.6%)	
June	pts	290 (8.4%)	17 (8.6%)	273 (8.4%)	0.660
July	pts	310 (8.9%)	19 (9.6%)	291 (8.9%)	
August	pts	274 (7.98%)	12 (6.1%)	262 (8.0%)	
September	pts	307 (8.9%)	14 (7.1%)	293 (9.0%)	
October	pts	280 (8.1%)	13 (6.6%)	267 (8.2%)	
November	pts	291 (8.4%)	17 (8.6%)	274 (8.4%)	
December	pts	295 (8.5%)	17 (8.6%)	278 (8.5%)	
Age	pts		57.2±15.6	56.3±15.6	0.416
Male	pts	2004 (57.9%)	117 (59.1%)	1887 (57.8%)	0.716
Weight	kg		78.7±17.1	77.6±15.8	0.423
BMI			26.8±5.0	26.8±4.9	0.966
NICU stay	day		15.3±11.7	4.8±5.4	<0.001
Admission					
Primary	pts	746 (21.5%)	47 (23.7%)	699 (21.4%)	
Secondary to 24 h	pts	739 (21.3%)	51 (25.8%)	688 (21.1%)	0.134
Secondary after 24 h	pts	1979 (57.1%)	100 (50.5%)	1879 (57.5%)	
Acute admission	pts	1020 (29.4%)	70 (35.4%)	950 (29.1%)	<0.001
Rehospitalisation	pts	40 (1.22%)	4 (2.0%)	44 (1.3%)	0.331
Diagnoses					
Stroke	pts	1498 (43.2%)	110 (55.6%)	1388 (42.5%)	
Trauma	pts	472 (13.6%)	27 (13.6%)	445 (13.6%)	
Tumour	pts	1078 (31.1%)	33 (16.7%)	1045 (32.0%)	<0.001
Epilepsy	pts	133 (3.8%)	3 (1.5%)	130 (4.0%)	
Hydrocephalus	pts	119 (3.4%)	13 (6.6%)	106 (3.2%)	
Infection	pts	88 (2.5%)	11 (5.6%)	77 (2.4%)	
Others	pts	75 (2.2%)	1 (0.5%)	74 (2.3%)	
Stroke	pts				<0.001
Ischemic	pts	580 (16.7%)	21 (10.6%)	559 (17.1%)	
ICH	pts	471 (13.6%)	49 (24.7%)	422 (12.9%)	
SAH	pts	447 (12.9%)	40 (20.2%)	407 (12.5%)	
TISS on admission			54.7±1.9	56.0±1.7	<0.001
TISS total			270632.8±231533.1	60415.1±92140..3	<0.001
GCS on admission			11.5±3.5	13.1±3.0	<0.001
APACHE II on admission			15.1±5.5	11.8±5.8	<0.001
GOS on NICU discharge			3.1±1.1	3.9±1.1	<0.001
Mortality in NICU	pts	152 (4.4%)	21 (10.6%)	131 (4.0%)	<0.001
Mortality in NICU	day		16.2±10.4	7.5±5.7	<0.001
CRP on admission			31.7±45.6	17.5±39.1	<0.001
CRP postoperative			30.0±44.4	14.0±33.0	<0.001
CRP 1 day after operation			59.8±56.9	31.6±39.6	<0.001
CRP highest in NICU stay			228.0±122.5	66.1±80.3	<0.001

*BMI* body mass index, *NICU* neurointensive care unit, *ICH* intracerebral haemorrhage, *SAH* subarachnoid haemorrhage, *TISS* Therapeutic Intervention Scoring System, *GCS* Glasgow Coma Scale, *APACHE* Acute Physiology and Chronic Health Evaluation, *GOS* Glasgow Outcome Scale, *CRP* C-reactive protein

Characteristics of brain operations can be seen in Table 2. Patients who had undergone operations and drainage had significantly higher nosocomial infection. These patients had more endotracheal tubes and tracheostomies, mechanical ventilations (Table 3), artery and central venous catheters (Table 4), urine and gastrointestinal tubes (Table 5).

**Table 2:** Characteristics of brain operations

Operation	Unit	Total population *N*=2231	NI group *N*=151	Control group *N*=2080	*p* value
Operation	pts	2231(64.4%)	151(76.3%)	2080 (63.7%)	<0.001
More than 1 operation	pts	214(9.6%)	42(27.8%)	172(8.3%)	<0.001
ASA score			3.8±1.0	3.1±1.1	<0.001
Day of hospitalisation	day		5.5±9.8	7.1±17.1	0.430
Day of NICU			1.6±1.3	1.3±1.1	0.535
Acute operation	pts	905(40.6%)	106(70.2%)	799(38.4%)	<0.001
Reoperation	pts	479(21.5%)	58(38.4%)	421(20.2%)	<0.001
Time of operation	minutes		151.9±108.4	137.7±89.4	0.080
Craniotomy	pts	1361(61.0%)	82(54.3%)	1279(61.5%)	0.080
Craniectomy	pts	363(16.3%)	50(33.1%)	313(15.0%)	<0.001
Trepanation	pts	227(10.2%)	23(15.2%)	204(9.8%)	0.033
Hypophysis	pts	85(3.8%)	0(0.0%)	85(4.1%)	0.011
Shunt	pts	108(4.8%)	12(7.9%)	96(4.6%)	0.066
Others	pts	99(4.4%)	9(6.0%)	90(4.3%)	0.347
Drainage	pts	1678(75.2%)	131(86.8%)	1547(74.4%)	<0.001
Redon	pts	858(38.5%)	49(32.5%)	809(38.9%)	0.001
Time overall	day		2.0±0.9	1.8±1.3	0.395
Gravity drainage	pts	807(36.2%)	75(49.7%)	732(35.2%)	0.029
Time overall	day		3.5±2.1	2.7±2.2	0.004
Lumbar	pts	218(9.8%)	36(23.8%)	182(8.8%)	<0.001
Day overall	day		7.7±5.5	5.1±3.2	<0.001
Ventricular	pts	138(6.2%)	21(13.9%)	117(5.6%)	<0.001
Day overall	day		13.4±9.9	5.9±4.3	<0.001

*ASA* American Society of Anesthesiologists, *NICU* neurointensive care unit

**Table 3:** Characteristics of respiratory procedures

Parameter	Unit	Total population *N*=3646	NI group *N*=198	Control group *N*=3266	*p* value
Airways	pts	710 (20.5%)	112 (56.6%)	598 (18.3%)	<0.001
ETT	pts	327(46.1%)	15(13.4%)	312(52.2%)	
TSK	pts	161(22.7%)	29(25.9%)	132(22.1%)	<0.001
ETT/TST	pts	222(31.3%)	68(60.7%)	154(25.8%)	
ETK time NICU	day		4.2±2.1	2.9±2.2	<0.001
ETK time	day		4.4±2.1	2.9±2.3	<0.001
TSK time NICU	day		14.2±10.2	8.4±7.8	<0.001
TSK time	day		21.8±34.6	21.6±62.2	0.980
TSK type Classic	pts	43(11.2%)	9(9.3%)	34(11.9%)	0.456
TSK NICU made	pts	250(65.3%)	75(77.3%)	175(61.2%)	0.006
Mechanical ventilation	pts	543(15.7%)	87(43.9%)	456(14.0%)	<0.001
Invasive	pts	539(99.3%)	87(100.0%)	452(99.1%)	<0.001
Time	day		14.1±9.9	5.6±5.9	<0.001
Indication					
Neuro	pts	414(76.2%)	54(62.1%)	360(78.9%)	0.161
Respiratory	pts	32(5.9%)	7(8.0%)	25(5.5%)	

*ETT* endotracheal tube, *TST* tracheostomy tube, *NICU* neurointensive care unit

**Table 4:** Characteristics of vascular catheters

Parameter	Unit	Total population *N*=3464	NI group *N*=198	Control group *N*=3266	*p* value
Artery catheter	pts	907(26.2%)	90(45.5%)	817(25.0%)	<0.001
Time	day		9.5±6.6	7.5±3.7	0.018
Number of artery catheters		923(100.0%)	91(100.0%)	832(100.0%)	
Radialis	pts	873(94.6%)	89(97.8%)	784(94.2%)	0.165
Brachialis	pts	14(1.5%)	0(0.0%)	14(1.7%)	0.211
Femoralis	pts	36(3.9%)	2(2.2%)	34(4.1%)	0.371
Left	pts	598(64.8%)	64(70.3%)	534(64.2%)	0.275
Time in NICU	day		8.27±5.45	4.10±3.36	0.094
Time all	day		8.41±5.40	4.41±3.43	0.377
Made in NICU	pts	216(23.4%)	47(51.6%)	169(20.3%)	<0.001
Made in operation theatre	pts	607(65.8%)	46(50.5%)	561(67.4%)	0.001
Cultivation of catheter	pts	691(74.9%)	74(81.3%)	617(74.2%)	0.157
Positive	pts	113(16.4%)	18(24.3%)	95(15.4%)	0.050
STSP	pts	100(88.5%)	13(72.2%)	87(91.6%)	0.018
Haemoculture cultivation	pts	164(17.8%)	31(34.1%)	133(16.0%)	<0.001
Positive	pts	34(20.7%)	9(29.0%)	25(18.8%)	0.206
STSP	pts	18(52.9%)	3(33.3%)	15(60.0%)	0.169
Central venous catheter	pts	372(10.7%)	64(32.3%)	308(9.4%)	<0.001
Time overall	day		9.9±7.4	7.5±3.7	0.077
Number of venous catheter		378(100%)	66(100%)	312(100%)	
Subclavia	pts	336(88.9%)	60(90.9%)	276(88.5%)	0.308
Jugularis	pts	19(5.0%)	1(1.5%)	18(5.8%)	0.157
Femoralis	pts	16(4.2%)	4(6.1%)	12(3.8%)	0.398
Axilaris	pts	7(1.9%)	1(1.5%)	6(1.9%)	0.836
Right	pts	323(85.4%)	59(89.4%)	264(84.6%)	0.164
Type one-line	pts	75(19.8%)	10(15.2%)	65(20.8%)	
Type two-line	pts	192(50.8%)	39(59.1%)	153(49.0%)	0.214
Type three-line	pts	64(16.9%)	8(12.1%)	56(17.9%)	
Time in NICU	day		8.20±7.31	4.70±4.92	<0.001
Time all	day		11.19±8.70	7.24±5.50	<0.001
Made in NICU	pts	162(42.9%)	41(62.1%)	121(38.8%)	<0.001
Made in operation theatre	pts	14(3.7%)	1(1.5%)	13(4.2%)	0.309
Cultivation of catheter	pts	261(69.0%)	45(68.2%)	216(69.2%)	0.977
Positive	pts	52(19.9%)	16(35.6%)	36(16.7%)	0.004
STSP	pts	40(76.9%)	10(62.5%)	30(83.3%)	0.010
Haemoculture cultivation	pts	72(19.0%)	16(24.2%)	56(17.9%)	0.090
Positive	pts	15(20.8%)	2(12.5%)	13(23.2%)	0.352
STSP	pts	13(86.7%)	2(100.0%)	11(84.6%)	0.551

*NICU* neurointensive care unit, *STSP* Staphylococcus species

**Table 5:** Characteristics of urine and gastrointestinal procedures

Parameter	Unit	Total population *N*=3464	NI group *N*=198	Control group *N*=3266	*p* value
Urine catheter	pts	3166(91.4%)	189(95.5%)	2927(89.6%)	0.008
Epicystostomy	pts	6(0.2%)	1(0.5%)	5(0.2%)	0.247
Time	day		15.5±11.6	4.7±5.5	<0.001
Time overall	day		22.6±13.1	12.8±9.7	<0.001
Gastrointestinal tube	pts	904(26.1%)	128(64.6%)	776(23.8%)	<0.001
Nasogastric tube	pts	882(25.5%)	125(63.1%)	757(23.2%)	<0.001
Time	day		15.4±11.2	6.2±6.9	<0.001
Time overall	day		19.6±12.6	10.7±9.4	<0.001

We confirmed transfusions (*p*<0.001), ulcer prophylaxis (*p*<0.001) and corticoids (*p*=0.002) as further parameters influencing nosocomial infection, but we did not see more nosocomial infection in patients with diabetes mellitus (*p*=0.203), (Table 6).

**Table 6:** Further monitored parameters influencing onset of nosocomial infection

Parameter	Unit	Total population *N*=3464	NI group *N*=198	Control group *N*=3266	*p* value
Corticoids	pts	1172(33.8%)	47(23.7%)	1125(34.4%)	0.002
Dexamethasone	pts	944(27.3%)	31(15.7%)	913(28.0)	<0.001
Methylprednisolone	pts	35(1.0%)	5(2.5%)	30(0.9%)	0.028
Hydrocortisone	pts	241(7.0%)	12(6.1%)	229(7.0%)	0.610
Time	day		6.37±8.78	3.58±2.56	<0.001
Transfusions	pts	176(5.1%)	41(20.7%)	135(4.1%)	<0.001
Number			2.46±8.78	2.57±2.56	0.695
Blood loss	ml		523.77±668.07	380.74±478.76	0.019
Haemoglobin			93.35±21.03	115.34±21.62	<0.001
Ulcer prophylaxis	pts	1838(53.1%)	134(67.7%)	1704(52.2%)	<0.001
One medicine	pts	1669(48.2%)	119(60.1%)	1550(47.5%)	0.406
Sucralfate	pts	758(21.9%)	26(13.1%)	732(22.4%)	0.002
H2 antagonist	pts	196(5.7%)	27(13.6%)	169(5.2%)	<0.001
Omeprazole	pts	1062(30.7%)	97(49.0%)	965(29.5%)	<0.001
Diabetes Mellitus	pts	491(14.2%)	22(11.1%)	469(14.4%)	0.203
Op. wound complication	pts	133(3.8%)	35(17.7%)	98(3.0%)	<0.001
Liquorrhoea	pts	81(2.3%)	23(11.6%)	58(1.8%)	<0.001

ESBL occurred in 1.9% and MRSA in 1.5% of the total population, without differences between NI group patients and the control group (Table 7). We did not have any case of vancomycin-resistant enterococcus.

**Table 7:** Multidrug-resistant bacteria ESBL and MRSA in NICU

Parameter	Unit	Total population *N*=3464	NI group *N*=198	Control group *N*=3266	*p* value
Multidrug-resistant	pts	116(3.3%)	12(6.1%)	104(3.2%)	0.029
ESBL	pts	67(1.9%)	6(3.0%)	61(1.9%)	0.566
On admission	pts	36(1.0%)	4(2.0%)	32(1.0%)	0.249
Nose	pts	11(0.3%)	1(0.5%)	10(0.3%)	0.986
Throat	pts	21(0.6%)	4(2.0%)	17(0.5%)	0.051
Trachea	pts	15(0.4%)	1(0.5%)	14(0.4%)	0.725
Urine	pts	19(0.5%)	0(0.0%)	19(0.6%)	0.106
Rectum	pts	31(0.9%)	3(1.5%)	28(0.9%)	0.848
Brain	pts	2(0.1%)	1(0.5%)	1(0.0%)	0.039
Others	pts	5(0.1%)	1(0.5%)	4(0.1%)	0.369
MRSA	pts	52(1.5%)	7(3.5%)	45(1.4%)	0.320
On admission	pts	22(0.6%)	0(0.0%)	22(0.7%)	0.015
Nose	pts	27(0.8%)	4(2.0%)	23(0.7%)	0.766
Throat	pts	11(0.3%)	1(0.5%)	10(0.3%)	0.632
Trachea	pts	14(0.4%)	2(1.0%)	12(0.4%)	0.916
Brain	pts	5(0.1%)	1(0.5%)	4(0.1%)	0.652
Haemoculture	pts	1(0.0%)	0(0.0%)	1(0.0%)	0.690
Others	pts	5(0.1%)	0(0.0%)	5(0.2%)	0.354

*NICU* neurointensive care unit, *ESBL* Extended spectrum beta-lactamase, *MRSA* Methicillin-resistant Staphylococcus aureus

Antibiotics policy is shown in Table 8. Antibiotic prophylaxis was given to 63% of the total population, mostly (59.2%) in association with operations. In 33.4% of the patients it was only administered in the operating theatre. Prolonged administration in the NICU was associated with more NIs (*p*=0.017). Antibiotic therapy was given to 9.7% of the total population.

**Table 8:** Administration of antibiotics in NICU

Parameter	Unit	Total population *N*=3464	NI group *N*=198	Control group *N*=3266	*p* value
Antibiotic prophylaxis	pts	2183(63.0%)	127(64.1%)	2056(63.0%)	0.736
One prophylaxis	pts	1931(55.7%)	91(46.0%)	1840(56.3%)	<0.001
Operation	pts	2049(59.2%)	116(58.6%)	1933(59.2%)	0.222
Only operation theatre	pts	1157(33.4%)	61(30.8%)	1096(33.6%)	
Operation 1 dose	pts	924(26.7%)	42(21.2%)	882(27.0%)	
Operation 2 doses	pts	191(5.5%)	14(7.1%)	177(5.4%)	0.006
Operation 3 doses	pts	40(1.2%)	4(2.0%)	36(1.1%)	
Operation 4 doses	pts	2(0.1%)	1(0.5%)	1(0.0%)	
NICU	day		4.96±5.69	3.31±2.88	0.017
Others					
Aspiration	pts	51(1.5%)	5(2.5%)	46(1.4%)	0.218
Suspected infection	pts	49(1.0%)	2(1.0%)	47(1.4%)	0.600
Trauma	pts	30(1.4%)	2(1.0%)	28(0.9%)	0.844
Liquorrhoea	pts	46(0.9%)	6(3.0%)	40(1.2%)	0.034
Drainage	pts	35(1.3%)	6(3.0%)	29(0.9%)	0.004
Others	pts	31(1.0%)	4(2.0%)	27(0.8%)	0.090
NICU	Day		7.75±4.61	4.54±3.33	<0.001
Type of antibiotic					
Cefazolin	pts	1733(50.0%)	106(53.5%)	1627(49.8%)	0.242
Amoxicillin clavulanate	pts	362(10.5%)	30(15.2%)	332(10.2%)	0.028
Clindamycin	pts	127(3.7%)	5(2.5%)	122(3.7%)	0.351
Antibiotic therapy	pts	335(9.7%)	169(85.4%)	166(5.1%)	<0.001
One infection	pts	326(9.4%)	161(81.3%)	165(5.1%)	0.019
One antibiotic	pts	220(6.4%)	100(50.5%)	120(3.7%)	0.061
Two antibiotics	pts	78(2.3%)	44(22.2%)	34(1.0%)	
NICU start	pts	224(6.5%)	151(76.3%)	73(2.2%)	<0.001
Empirical therapy	pts	201(5.8%)	101(51.0%)	100(3.1%)	0.929
According to cultivation	pts	189(5.5%)	106(53.5%)	83(2.5%)	0.019
Days of ATB all	day		8.82±6.89	6.09±4.95	<0.001
Type of antibiotic					
Ceftriaxone	pts	34(1.0%)	9(4.5%)	25(0.8%)	0.003
Ceftazidime	pts	6(0.2%)	3(1.5%)	3(0.1%)	0.982
Meropenem	pts	75(2.2%)	48(24.2%)	27(0.8%)	0.008
Penicillin	pts	13(0.4%)	5(2.5%)	8(0.2%)	0.378
Oxacillin	pts	23(0.7%)	17(8.6%)	6(0.2%)	0.020
Ciprofloxacin	day	84(2.4%)	57(28.8%)	27(0.8%)	<0.001
Trimethoprim	pts	17(0.5%)	10(5.1%)	7(0.2%)	0.478
Gentamicin	pts	25(0.7%)	15(7.6%)	10(0.3%)	0.321
Others	pts	71(2.0%)	29(14.6%)	42(1.3%)	0.068

*NICU* neurointensive care unit, *ATB* antibiotic

We compared patients with NI onset in the NICU (77.3%) with NI present on admission (22.7%), (Table 9). We identified 153 (4.4%; wound 1.0%, respiratory 1.7%, urinary 0.9%, bloodstream 0.6% and other 0.1%) patients with NI onset in the NICU. Patients with NI onset in the NICU stayed in the NICU significantly longer, and were more expensive, but these patients did not have higher mortality. Multivariate logistic regression analysis seeking significant predictors for onset of NI in the NICU can be seen in Table 10. Our results showed that strong predictors on onset of NI in our neurocritical care were accesses such as airways and urine catheters, NICU stay, antibiotic prophylaxis, wound complications and transfusion. This analysis did not find the multidrug-resistant bacteria as ESBL and MRSA to be a predictor of NI.

**Table 9:** Nosocomial infections on admission and onset in the NICU

Parameter	Unit	NI total	NI on admission	NI onset in NICU	*p* value
Number total	pts	198 (100%)	45 (22.7%)	153 (77.3%)	
Age	pts	57.2±15.6	53.7±16.9	58.3±15.1	0.086
Male	pts	117(59.1%)	18(40.0%)	63(41.2%)	<0.001
NICU stay	day	15.3±11.7	6.9±7.2	17.7±11.6	<0.001
Diagnoses					
Stroke	pts	110(55.6%)	13(28.9%)	97(63.4%)	
Trauma	pts	27(13.6%)	3(6.7%)	24(15.7%)	
Tumour	pts	33(16.7%)	13(28.9%)	20(13.1%)	
Epilepsy	pts	3(1.5%)	0(0.0%)	3(2.0%)	<0.001
Hydrocephalus	pts	13(6.6%)	7(15.6%)	6(3.9%)	
Infection	pts	11(5.6%)	9(20.0%)	2(1.3%)	
Others	pts	1(0.5%)	0(0.0%)	1(0.7%)	
TISS on admission		54.7±1.9	56.0±179	54.3±1.8	<0.001
TISS total		270632.8±231533.1	111173.7±231533.1	309492.6±234698.9	<0.001
GCS on admission		11.5±3.5	12.0±3.3	11.3±3.5	0.234
APACHE II on admission		15.1±5.5	13.6±5.4	15.4±5.5	0.099
GOS on NICU discharge		3.1±1.1	3.5±1.2	3.0±1.1	0.015
Mortality in NICU	pts	21(10.6%)	3(6.7%)	18(11.8%)	0.329
Operation	pts	151(76.3%)	37(82.2%)	114(74.5%)	0.285
Airways	pts	112(56.6%)	16(35.6%)	96(62.7%)	0.001
Mechanical ventilation	pts	87(43.9%)	7(15.6%)	80(52.3%)	<0.001
Artery catheter	pts	90(45.5%)	6(13.3%)	84(54.9%)	<0.001
Central venous catheter	pts	64(32.3%)	11(24.4%)	53(34.6%)	0.199
Lumbar drainage	pts	36(18.2%)	5(11.1%)	31(20.3%)	0.162
Ventricular drainage	pts	21(10.6%)	3(6.7%)	18(11.8%)	0.329
Corticoids	pts	47(23.7%)	11(24.4%)	36(23.5%)	0.899
Transfusions	pts	41(20.7%)	5(11.1%)	36(23.5%)	0.071
Ulcer prophylaxis	pts	134(67.7%)	27(60.0%)	107(69.9%)	0.210
Diabetes Mellitus	pts	22(11.1%)	3(6.7%)	19(12.4%)	0.280
Antibiotic prophylaxis	pts	127(64.1%)	23(51.1%)	104(68.0%)	0.038
Antibiotic therapy	pts	169(85.4%)	28(62.2%)	141(92.2%)	<0.001
ESBL	pts	6(3.0%)	1(2.2%)	5(3.3%)	0.719
MRSA	pts	7(3.5%)	1(2.2%)	6(3.9%)	0.587
One infection	pts	189(95.5%)	45(100.0%)	144(94.1%)	
Two infections	pts	8(4.0%)	0(0.0%)	8(5.2%)	0.250
Three infections	pts	1(0.5%)	0(0.0%)	1(0.7%)	
Bloodstream	pts	23(11.6%)	1(2.2%)	22(14.4%)	0.025
Vascular catheter	pts	14(7.1%)	1(2.2%)	13(8.5%)	0.149
Respiratory	pts	63(31.8%)	3(6.7%)	60(39.2%)	< 0.001
VAP	pts	34(17.2%)	1(2.2%)	33(21.6%)	0.002
Urinary	pts	35(17.7%)	5(11.1%)	30(19.6%)	0.189
Urinary catheter	pts	33(16.7%)	5(11.1%)	25(16.3%)	0.255
Wound without operation	pts	2(1.0%)	1(2.2%)	1(0.7%)	0.355
Wound with operation	pts	70(35.4%)	35(77.8%)	35(22.9%)	<0.001
Wound complication					
Liquorrhoea	pts	14(7.1%)	7(15.6%)	7(4.6%)	0.012
Dehiscence	pts	11(5.6%)	9(20.0%)	2(1.3%)	<0.001
Fistula	pts	6(3.6%)	3(6.7%)	3(2.0%)	0.105

*NICU* neurointensive care unit, *TISS* Therapeutic Intervention Scoring System, *GCS* Glasgow Coma Scale, *APACHE* Acute Physiology and Chronic Health Evaluation, *GOS* Glasgow Outcome Scale, *ESBL* Extended spectrum beta-lactamase, *MRSA* Methicillin-resistant Staphylococcus aureus, *VAP* ventilator associated pneumonia

**Table 10:** Multivariate logistic regression analysis of nosocomial infection onset in NICU

Multivariate analysis				
Nosocomial infections predictors	Odds Ratio	Lower CL 95%	Upper CL 95%	*p* value
NICU stay (per day)	1.14	1.12	1.16	< 0.001
Airways	2.69	1.81	3.99	< 0.001
Urine catheter	2.77	1.00	7.70	0.050
Transfusions	1.79	1.07	2.97	0.025
Wound complications	2.30	1.33	3.97	0.003
Antibiotic prophylaxis	0.50	0.34	0.74	< 0.001

*NICU* neurointensive care unit, *CL* confidence limit

## Discussion

Maintaining nosocomial infection control management is one marker of quality in neurocritical care. Its target is to improve clinical outcomes and decrease costs in the neurocritical care unit. Preventions of nosocomial infections are an important issue in all medical or surgical critical care units, but in neurocritical care they have an additional risk as a cause of secondary brain damage, which affects the morbidity and mortality of primary brain diseases [1–5]. As the aim of neurocritical care is to avoid all insults causing secondary brain damage, preventive management of nosocomial infections is a challenge for neurointensivists. Incidence of nosocomial infections can be reduced by keeping a hygienic and epidemiological regime and rational antibiotic policy. Nosocomial infection management demands constant maintenance and stable teamwork while maintaining standard procedures. We present our preventive multimodal nosocomial infection protocol, which we implemented in our NICU. The first phase involves imposing hygienic principles and the antibiotics policy. The second phase, actually keeping to this protocol, is a much more difficult task in our experience, as a vital component for its success is the participation of the whole team, from doctors and nurses to cleaners working in the neurocritical care unit and even visitors. The use of standard procedures and meticulous checks are an important part of the regime.

Here we present the impact of our preventive nosocomial infection management on the incidence of nosocomial infections in all the patients admitted to our NICU with acute brain disease. The results show that our preventive protocol was not sufficient to completely eliminate all nosocomial infections, but it did lead to a relatively low nosocomial infection incidence of 4.4%. We did not observe differences between various seasons of the year, either among primary or secondary admissions, but we did among acute admissions, acute operations and reoperations. Infections were more frequently associated with strokes than other brain diagnoses. There were significantly more infections in airways, mechanical ventilations and catheters, but only airways and urine catheters were strong predictors in multivariate logistic regression analysis. These are still risk factors which remained despite the maintenance of the preventive strategy. Further predictors were confirmed to be the well-known factors of NICU stay, wound complications, antibiotic prophylaxis and transfusion.

The increasing colonisation of multidrug-resistant bacteria ESBL and MRSA is a big problem among critically ill patients and this situation is getting worse. At present, many patients already have these bacteria on admission and this colonization constitutes a risk of nosocomial infections [16–18]. We deal with this by completely isolating these patients using barrier care techniques in order to prevent the transmission of these multidrug-resistant ESBL and MRSA to other, uncolonised patients. This was reflected in our results, which showed that we had newly occurred ESBL in only in 31 (0.9%) patients and MRSA in 30 (0.9%) patients. In this study we did not find that multidrug-resistant bacteria were a predictor of nosocomial infections.

Antibiotics policy, predominantly the overuse of antibiotics, is another big issue in preventive multimodal nosocomial infection protocol. From our results, we see that antibiotic prophylaxis is mainly used in association with operations and only 9.7% of the total population received antibiotic therapy. Unindicated use of antibiotics contributes to the emergence and spread of multidrug-resistant bacteria, which are becoming a growing problem in healthcare facilities. Antibiotics should only be given during operations and their administration should not be prolonged in the NICU. During the prophylactic use of antibiotics it is essential not only to keep to the indication, but also to maintain the time of administration. However, this study confirmed that antibiotic prophylaxis policy is an important task, because antibiotic prophylaxis was found to be a predictor of nosocomial infection in the neurocritical care population. While using antibiotics, it is essential to maintain the correct administration and not use antibiotics during the colonisation of the patient, but only for the infection. Timing, dosage and tissue penetration are important in their administration.

Our microbiological screening was the same for all patients, who can therefore be compared easily. The unified system included nose, throat, trachea, skin, urine and rectum tests from admission, so that we would know what the patient was admitted with, and then regularly every three days. This means that this microbiological screening sometimes fell on the weekend, which at first was difficult to implement in the microbiological department. Regular microbiological screening from admission took place every three days, giving us an overview of the microbiological state of the patient and allowing us to find colonization of multidrug-resistant bacteria [18] and further perform the targeted antibiotic treatment of nosocomial infections.

Although it would be better to have single-patient boxes, the lay-out of four divided rooms provides some of the benefits and enables the isolation of patients with multidrug-resistant bacteria ESBL and MRSA, as it is very important to isolate these patients so that these bacteria do not spread to the rest of the NICU and the other patients. Our results show that over a ten-year period we did not have a large incidence of the multidrug-resistant bacteria ESBL and MRSA, while there was not a single case of VRE. This is in contrast to the Minhas [19] study, where he mentioned 2.5% of VRE in the neurosurgical and neurological intensive care unit.

This study confirmed that accesses are still a risk factor for nosocomial infection. Due to increasing numbers of invasive medical procedures in neurocritical care, local preventive infection control management has an important task. Although preventive multimodal strategy is widely known to reduce nosocomial infection and multidrug resistant bacteria, it is sometimes difficult to maintain. Nonetheless, the results of this study show the importance of this maintenance. We present our 10 year prospective infection control management, which was efficient, as it led to a rate of 4.4% nosocomial infections in acute neurological and neurosurgical care patients. Due to multiple testing, there is a higher probability of family-wise error. On the other hand, the results must be read in context, not every p-value below 0.05 is commented on as a finding.

This study showed prospective infection control management in 3464 neurocritically care patients. Although they all came from a single neurocentre, which is a limitation of this study, there are already many more epidemiologic studies regarding nosocomial infection control and multi-drug resistant bacteria from the medical and surgery intensive care units than from neurocritical care units, whether neurosurgical or neurological, and very few studies concerned with neurological-neurosurgical critical care units [19, 20]. In this area, more studies focus on specific diagnoses [1, 2, 7, 21, 22] than whole neurocritical care populations.

## Conclusions

This study showed that this preventive multimodal nosocomial infection control management was efficient, because it gave low rates of nosocomial infections (4.2%), both ESBL and MRSA in a mere 0.9% of patients each and not a single case of VRE. Strong predictors for the onset of nosocomial infections were accesses such as airways and urine catheters, NICU stay, antibiotic prophylaxis, wound complications and transfusion. This study confirmed the well-known fact that nosocomial infections are associated with worse outcome, higher cost and longer NICU stay.

## Abbreviations

APACHE: Acute Physiology and Chronic Health Evaluation
ASA: American Society of Anesthesiologists
ATB: antibiotic
BMI: body mass index
CRP: C-reactive protein
ESBL: Extended spectrum beta-lactamase
ETT: endotracheal tube
GCS: Glasgow Coma Scale
GOS: Glasgow Outcome Scale
ICH: intracerebral haemorrhage
MRSA: Methicillin-resistant Staphylococcus aureus
NI: nosocomial infection
NICU: neurointensive care unit
SAH: subarachnoid haemorrhage
STSP: *Staphylococcus species*
TISS: Therapeutic Intervention Scoring System
TST: tracheostomy tube
VRE: Vancomycin-resistant enterococcus
